# Marketing of Food and Beverages to Children in the Eastern Mediterranean Region: A Situational Analysis of the Regulatory Framework

**DOI:** 10.3389/fnut.2022.868937

**Published:** 2022-05-18

**Authors:** Ayoub Al-Jawaldeh, Jana Jabbour

**Affiliations:** ^1^Regional Office for the Eastern Mediterranean, World Health Organization, Cairo, Egypt; ^2^Department of Nutrition, School of Health Sciences, Modern University for Business and Sciences, Beirut, Lebanon

**Keywords:** Eastern Mediterranean Region, marketing, childhood obesity, media, unhealthy food

## Abstract

Marketing of food items high in added saturated and/or trans-fat, sugar, or sodium (HFSS) negatively affect consumption patterns of young children. The World Health Organization (WHO) advised countries to regulate the marketing of foods and non-alcoholic beverages to young populations. The aim of this manuscript is to provide a situational analysis of the regulatory framework of food marketing policies targeting children in the Eastern Mediterranean Region (EMR). A semi structured questionnaire was shared with the focal points of EMR member states inquiring about the reforms and monitoring initiatives in place. Electronic databases were searched for relevant publications between 2005 and 2021. Results revealed that even though 68% of countries discussed the recommendations, progress toward the WHO set goals has been slow with only 14% of countries implementing any kind of restrictions and none executing a comprehensive approach. Reforms have focused on local television and radio marketing and left out several loopholes related to marketing on the internet, mobile applications, and cross border marketing. Recent monitoring initiatives revealed a slight improvement in the content of advertised material. Yet, unhealthy products are the most promoted in the region. This review identified the need to intensify the efforts to legislate comprehensive food marketing policies within and across EMR countries.

## Graphical Abstract

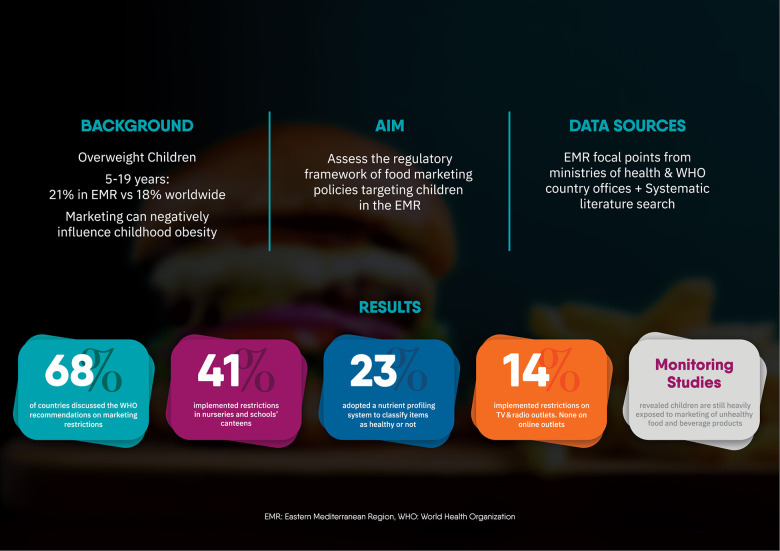

## Introduction

**FIGURE 1:**
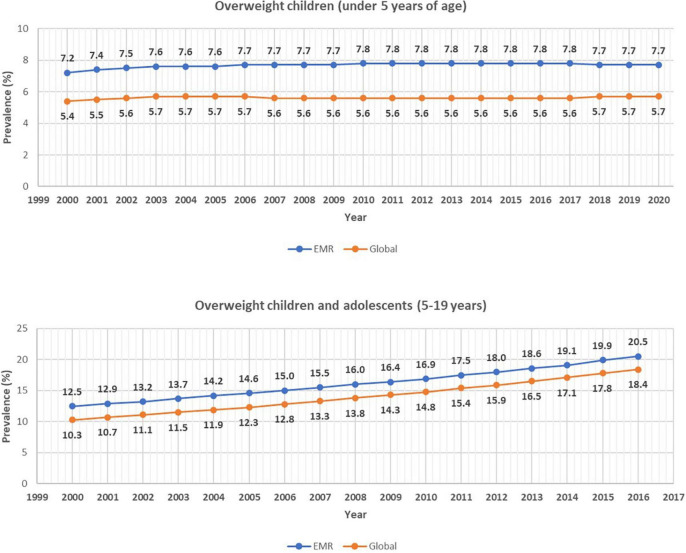
Prevalence of overweight children under five years (top panel) and aged 5–19 years (bottom panel) in the Eastern Mediterranean Region (EMR) and globally. Overweight was defined as % weight for height 2 standard deviations above median for children under 5 years of age and as 1 standard deviation above median body mass index for age for children and adolescents aged 5–19 years. Source: Numbers have been extracted from the Global Health Observatory [5, 6].

The Eastern Mediterranean Region (EMR) has been facing many health challenges due to unhealthy lifestyle patterns, political instability, and fragile health systems (1, 2). As a result, chronic diseases have increased from 6% in 1980 to 14% in 2014 and the burden of obesity on Disability-Adjusted Life-Years (DALYs) grew from 4% in 1990 to 8% in 2013 (3, 4). Obesity has been weighing heavily on children in the region. Compared to 5.7% of children under five around the world, 7.7% of EMR children were overweight or obese in 2020 (5, 6) (Figure 1). Similarly, the prevalence rate of overweight and obesity for children and adolescents aged 5-19 years in 2016 was 20.5% in the EMR compared to 18.4% worldwide (5) (Figure 1). The EMR has five [Kuwait, Kingdom of Saudi Arabia (KSA), Qatar, Oman, Libya] and two (Oman and Iran) out of the ten countries in the world with the highest prevalence and relative change in overweight children aged 2–4, respectively (7). Malnutrition manifestations differ based on the countries’ Human Development Index, progress in nutrition transition, political stability, environmental status, mean population age, etc. (3, 8). Low- and middle-income countries have been struggling with the “double burden” of malnutrition with co-existence of wasting and obesity within the same population (9, 10).

Children, starting from a young age, are exposed to food marketing at home, nurseries, schools, and in public spaces, most of which are for products high in added saturated and/or trans fatty acids, sugar and sodium (HFSS) (11, 12). Marketing of food and beverages influences children’ knowledge of products, preferences, acquisitions, and dietary patterns (13–16). In 2004, the World Health Organization (WHO) called on the private sector to adopt a responsible marketing approach toward children (17). Beyond corporate social responsibility, the need for legal reforms to enhance dietary patterns has been highlighted recurrently (18, 19). In 2010, the World Health Assembly (WHA) adopted the WHO Set of Recommendations on Marketing of Foods and Non-alcoholic Beverages to Children (WHO Recommendations) and called on all countries to integrate policies to regulate the marketing of unhealthy foods to young populations (20). Best practice policies to protect children up to 18 years from the harmful impact of food marketing include legislation of all relevant foods, cover all forms of marketing and are robustly monitored and enforced with meaningful sanctions (21). A WHO report assessed the implementation of marketing restriction in the EMR up to 2018 and found a limited integration of reforms in the legal framework of the EMR region (22). This project is timely in view of the scarcity of scholarly articles assessing the progress of the EMR countries in implementing the WHO Recommendations. The aim of this paper is to provide an updated situational analysis of the progress of EMR countries in integrating a regulatory framework of food marketing policies targeting children.

## Materials and Methods

### Data Sources: Questionnaire and Search Strategy

A semi-structured questionnaire evaluating countries’ progress toward the WHA recommendations was administered on all EMR country representatives (selected from the countries ministries of health and/or the WHO country offices). The questionnaire was shared by email via the WHO EMR office in December 2021 in Arabic and English languages (English version available in Supplementary Material). It inquired from country representatives if restrictions on marketing of unhealthy food and beverage items to children (up to 18 years old) have been discussed and/or implemented, on the presence of voluntary pledges by the food industry, on the stakeholders involved in the relevant discussions and legislations, and on the monitoring initiatives undertaken by governmental and/or non-governmental bodies. The extent of the implementation of a recommendation/discussion/legislation across the region was calculated by dividing the number of countries which implemented a recommendation by the total number of member states in the EMR region (*n* = 22). Results were rounded to the nearest integer. Moreover, a systematic literature search was implemented on the following databases: Medline, PubMed, Scopus, Embase, Google Scholar, Al Manhal, Arab World Research Source, E-Marefa, and Iraqi Academic Scientific Journals. The last four databases were chosen as they provide data sources that are specific to the region, since Arabic is the language most spoken in EMR countries. Manual search was conducted on the websites of the WHO and Food and Agriculture Organization. Inclusion criteria included manuscripts, governmental documents, reforms, and policy briefs in countries of the EMR. The following terms were used: “Eastern Mediterranean Region” countries OR “Middle East” OR each state alone AND “Children” OR “Child” OR “Toddler” AND “Diet” OR “Diet Therapy” OR “Nutrient” OR “Consumption” OR “Saturated Fat” OR “Trans Fat” OR “Sodium” OR “Salt” OR “Sugar” OR “Juice” OR “Sugar Sweetened Beverages” OR “Soft Drinks” OR “Obesity” OR “Overweight” AND “Marketing” OR “Promotion” OR “Advertisements” OR “Policy” OR “Reform” OR “Pledge.” The literature search was restricted to the Arabic, English and French languages and to publications between January 1, 2005, until November 7, 2021. Individual EMR countries included in the search were selected based on the WHO country categorization: Afghanistan, Bahrain, Djibouti, Egypt, Iran (Islamic Republic of), Iraq, Jordan, KSA, Kuwait, Lebanon, Libyan Arab Jamahiriya, Morocco, Oman, Occupied lands of Palestine, Pakistan, Qatar, Somalia, Sudan, Syrian Arab Republic, Tunisia, United Arab Emirates (UAE), and Yemen (23).

### Food Marketing Regulatory Framework: Defining Concepts

In order to understand this situational analysis, we define in this section a few concepts relevant to the food marketing regulatory framework. The effectiveness of marketing is a function of two elements: the exposure of children to the marketing message and the power of the communication. The WHO defines power in marketing as the “content, design and creative strategies used to target and persuade” (20). Marketing is not limited to advertisements. It incorporates promotion on several media such as television, radio, billboard, magazine, packaging design, point of sale, and digital media, including mobile applications, “advergames” and blogs.

The industry and the government are the main regulatory actors that design and implement food marketing restrictions. Regulation can be done in a voluntary approach when the industry initiates and monitors the restrictions or in a mandatory manner when governmental entities design the policies and implement judiciary actions (24). Comprehensive and stepwise approaches are two commonly employed methods for integrating legal reforms. While the former method is more inclusive, the latter may be more practical to stakeholders as it progresses in phases by gradually incorporating additional outlets, food and beverage items, marketing mediums and/or settings (25). The WHO favors the comprehensive approach as the stepwise method may leave loopholes in the regulatory framework that the food industry can benefit from (26). Yet, if the comprehensive approach is not deemed feasible, the initiation of a stepwise approach is favored over the absence of any implementation (26).

Nutrient profiling is the practice of classifying a product as “healthy” or “unhealthy” based on its salt, fat and sugar content (27). Profiling facilitates the identification of products that need prohibition by legal reforms by laying a common framework for policy makers (28). The WHO office in the EMR adopted the European nutrient profiling model that assesses 18 food categories and identifies products with elevated HFSS (27).

## Health Reforms in the Eastern Mediterranean Region

### Legal Reforms

Table 1 provides an overview of the actions taken by EMR member states to prepare for the endorsement of the WHO Recommendations on marketing restrictions of unhealthy foods to children based on the questionnaire collected from country representatives. No information could be collected from the following countries: Djibouti, Libyan Arab Jamahiriya, and Somalia. Most countries (68%) discussed the WHO Recommendations on regulating marketing of unhealthy foods and beverages (Table 1). Afghanistan, Iraq, Syria, and Yemen reported not having discussed the Recommendations. Most countries that discussed the marketing restrictions did so after 2016. Ministries of health were the common stakeholder in the discussions and legal reforms. Other stakeholders included ministries of education, of trade and commerce, and municipalities and radio and television commissions. Voluntary pledges by the food industries were implemented in the KSA and UAE and less than a quarter of EMR countries (23%) adopted a nutrient profiling system (Table 1) (22).

**TABLE 1:** Summary of the actions taken by EMR countries to prepare and complement the implementation of legal reforms of marketing restrictions of unhealthy foods and beverages to children.

Countries	Discussed WHO recommendations	Stakeholders involved in discussions and legislation	Voluntary pledge by the private sector	Nutrient profiling system
Afghanistan	x	-	x	x
Bahrain	✓	Ministries: Health and Commerce and Trade	¥	x
Egypt	✓	Ministry of Health	x	x
Iran	✓	Undersecretary of Public Health; Ministries of Health and Culture and Islamic Guidance, Iran’s Standard Organization Iran’s Food and Drug Organization, Iran’s Medical Council, High council of Health and Nutrition Security, and the food industry	x	✓
Iraq	x	-	x	x
Jordan	✓	Ministries: Education, Health, Media and Industry, Trade and Supply. Jordan Food and Drug Administration.	x	x
KSA	✓	Ministries: Education, Health, Municipalities, Trade and Investment. Radio and Television Commission and audio-visual commission and the food industry	✓	✓
Kuwait	✓	Ministries: Health and Trade. Public Authority for Food and Nutrition	x	x
Lebanon	✓	Ministries of Health and Education	x	x
Morocco	✓	Stakeholders in the field of health, regulation, communication, education, research, agriculture, food industry, and civil society	x	✓
Oman	✓	Ministries: Health, Trade, and Municipalities	¥	x
Palestine	✓	Ministries: Health, Education, National Economy, Agriculture, Finance. Palestine Standards Institution, Higher Council for Youth, and Sport, Palestinian Food Industries Union. NGOs, bakeries	x	x
Pakistan	✓	Ministries: Health, Science and Technology. Provincial Food Authorities	x	x
Qatar	✓	Ministries: Commerce and Industry, Education and Higher Education, Finance, Municipality, Public Health. Health care centers, Qatar Olympic Committee, Qatar Diabetes Association, Universities, Ministry of Sports and Youth, Qatar Media Corporation, Customs General Authority, and the Food Industry	¥	✓
Sudan	✓	The Sudanese Standards and Metrology Organization, Federal Ministry of Health, Ministry of industry, Food security Technical Secretariat and private sector.	x	x
Syria	x	-	x	x
Tunisia	✓	Ministries: Health, Trade, Industry, Communication, Agriculture, Education, Social, Women, and Family. Representatives from the Civil Society.	x	Planned
UAE	✓	Ministries: Health and Prevention, Economy, Justice, Dubai Health Authority, Telecommunications and Digital Government Regulatory Authority, Department of Health—Abu Dhabi, National Media Council.	✓	✓
Yemen	x	Ministries: Health, Trade and Industry, Agriculture, Irrigation, Fish Wealth and Water and Environment and Local Administration	x	x

*x, absent; ✓, present; ¥, pledge signed but not implemented according to local focal points. KSA, Kingdom of Saudi Arabia; NGOs: Non-Governmental Organizations, UAE, United Arab Emirates.*

Table 2 presents an overview of the legislation and implementation of restrictions relevant to the WHO Recommendations. While 14% had legislation for media outlets, 41% of countries had restrictions in nurseries and schools’ canteens (Table 2). The mapping exercise revealed that no country implemented a comprehensive regulatory approach to limit marketing of unhealthy food and beverages to children on media outlets. Iran, KSA, Oman, Pakistan, Qatar, Tunisia, and the UAE started planning and/or implementing some levels of reforms. Iran adopted the widest level of reforms compared to other counties. The decision to ban all advertisements from kindergartens, schools, and public places where children are present was ruled in 1978 and reinforced in 2010. In 2009, it was prohibited to include children in advertisements. In 2016, a decision to ban promotion of food products during children’s TV and radio programs and to avoid featuring obese children in advertisements was taken. In 2020, a legislation targeting the general public identified 19 HFSS food products as unhealthy items that should not be marketed (22, 29, 30). Reforms included nutrient profiling and involved several governmental bodies such as the Ministry of Health, Ministry of Industry, National Standards Organization, and The Islamic Republic of Iran Broadcasting (22, 29). Yet, disrespect of these reforms has not been linked to any judiciary action. In Pakistan, provincial food authorities banned sale of soft drinks in and around teaching institutions. Following this decision, local media outlets took the initiative to stop advertising for unhealthy food and beverage items during children television programs. No relevant legislation has been passed in the country. In Oman, in compliance with the “Child Law,” the Ministry of Information passed legislation banning the promotion of food products during children’s programs on television and radio stations and prohibiting advertisement of printed material related to medical and pharmaceutical products without the approval of the Ministry of Health. Reforms in Pakistan and Oman have major loopholes, neither do they incorporate a step for nutrient profiling, nor do they include components of marketing on social media networks, roads, supermarkets, and restaurants. Moreover, the definitions of unhealthy food items in Pakistan differed based on the provinces with some limiting unhealthy items to “sugary foods and drinks” while others employed wider definitions that incorporated sodium rich snacks as well. Some countries like Morocco and UAE adopted nutrient profiling and legislated regulatory actions for marketing of unhealthy items to children but have not implemented relevant reforms yet. The UAE legislation provides general guidelines on the need to prohibit marketing of unhealthy foods to children but does not incorporate any implementation mechanism. Legislation was hence not considered present at this point (Table 2). Egypt, the KSA, Qatar and Tunisia have policy briefs currently under revision by their respective governments (Table 2). Whereas KSA and Qatar adopted nutrient profiling systems, Tunisia prepared one but has not adopted it yet and Egypt neither adopted nor implemented any system (Table 1) (31). Qatar’s proposed legislation involves a ban of children’s toy incentives in fast food restaurants, prohibition of advertising of unhealthy foods to children, regulation of the promotions of unhealthy foods in or around schools. Tunisia’s National Institute of Nutrition and Food Technology drafted a policy brief for restriction of marketing of food and beverages on television, on the internet and during sports and cultural festivities (22). Yet, the proposed policy does not impose restrictions on product packaging and on displaying cartoon characters on products, which may reduce its effectiveness.

**TABLE 2:** Summary of the legislation and restrictions implemented in canteens of educational facilities and on media outlets.

Countries	Legislation relevant to the WHO recommendations		Implementations of restrictions during children’s programs on media outlets		
	Media outlets	Nursery and schools’ canteens	Television	Radio	Social media networks
Afghanistan	x	x	x	x	x
Bahrain	x	✓	x	x	x
Egypt	Draft under review	x	x	x	x
Iran	✓	✓	✓	✓	x
Iraq	x	x	x	x	x
Jordan	x	✓	x	x	x
KSA	Draft under review	x	x	x	x
Kuwait	x	✓	x	x	x
Lebanon	x	x	x	x	x
Morocco	✓	x	x	x	x
Oman	¥	x	✓	✓	x
Palestine	x	✓	x	x	x
Pakistan	x	✓	✓	x	x
Qatar	Draft under review	✓	x	x	x
Sudan	x	x	x	x	x
Syria	x	x	x	x	x
Tunisia	Draft under review	x	x	x	x
UAE	x	✓	x	x	x
Yemen	x	✓	x	x	x

*x, absent; ✓, present; ¥, limited. KSA, Kingdom of Saudi Arabia; UAE, United Arab Emirates.*

Weak multisectoral collaboration is a challenge identified in the implementation of policies across the region. A recent scoping review revealed that regulation of marketing of food is weakly implemented in Iran due to weak scientific criteria in the legal reforms, lack of judiciary actions linked to violations, poor collaboration across sectors, and inadequate monitoring (30). Qualitative studies among stakeholders of childhood obesity in Iran criticized the weak coordination between stakeholders and the top-down approach in policy making. On the field stakeholders are poorly consulted, leading to weak implementation of these reforms (32, 33). In Pakistan, a content analysis of infant and children feeding policies identified the lack of clarity of the responsibilities of collaborators and poor stakeholders’ collaboration as the areas that need to be strengthened (34).

### Monitoring Initiatives

Governmental and academic bodies assessed the nature and quality of food marketing on media outlets in the EMR countries. A content analysis of the advertisements mostly viewed by a sample of 7–12-year-old children on local television channels in Egypt in 2015–2016 revealed that 74% of commercials were for unhealthy products such as sweets, chips and soft drinks (35). In Oman, a review of Pan-Arab TV stations popular among children and local radio and print media in 2015–2016 showed that print media advertisements rarely promoted unhealthy snacks but 71% of television and 44% of radio advertisements promoted HFSS items (36), with the majority of promotions being between children’s programs. Sweetened beverages were the most commonly sold products to students in stores around schools. These stores included promotions on HFSS products and featured cartoon characters for products at the point of sale (36). In Lebanon, an assessment of marketing on local television channels conducted in 2016–2017 revealed that 100 and 85% of commercials advertised during children’s programs and general audience programs were for unhealthy products, respectively. Moreover, around 80% of the commercials that included a health claim were for unhealthy food and beverages (37). An analysis of Iranian television commercials in 2016 revealed that the length of food and beverage advertisements was significantly shorter compared to previous years. Yet, 60% of television commercials remained food related, promoted items elevated in sugar and sodium, and favored sweetened fruit products over natural fruits (38). In KSA the Saudi Food and Drug Authority identified the YouTube channels that are most viewed by children in the country between years 2016 and 2021. A review of videos on these channels revealed that HFSS food items are commonly promoted through commercials, promotion codes, video characters consuming them prior to a competition, etc. (39). Moreover, a content analysis of printed and social media coverage of childhood obesity in the UAE revealed that excess weight among children was commonly presented as the result of bad parents’ choices. The influence of structural elements related to policies and the role of the food and beverage industry was found to be minimized in the media (40).

### Self-Regulation of the Food Industry

Prior to the governments’ policy changes, the food industry had responded to a call by the WHO in 2004 to regulate food marketing. Eight global food and beverage companies in countries of the Gulf Country Cooperation signed the Responsible Food and Beverage Marketing to Children Pledge in 2010 (41). Through this pledge, companies committed to market products deemed healthy to children under 12 years of age and to stop sales of unhealthy products in primary schools. This pledge was further strengthened in 2016 with companies harmonizing the evaluation criteria employed (42). In 2020, the International Food and Beverage Alliance’s (IFBA) compliance monitoring report to the pledge in the KSA and UAE revealed a 100% adherence rate with food marketing on television, printed material and the internet (43). Yet, these pledges and monitoring initiatives have been criticized for leaving major loopholes (22). While the IFBA pledges cover radio, television, company owned websites, cinemas, and mobile marketing, they exclude points of sale marketing, sponsorship of pediatric activities and are limited to children’s programs (22). Since children tend to watch programs that are not specifically for their age group, to access websites other than those owned by companies’ websites, and to attend to public spaces that do not have marketing restrictions, the latter monitoring studies can underestimate the rate of children exposure to the marketing of unhealthy products (22, 44). Moreover, the IFBA pledge limits its scope to children under 12 years of age and leaves out the age group of 12–18 years who is also much affected by marketing of HFSS (45).

## Discussion

### Marketing of Unhealthy Food and Beverages in the Eastern Mediterranean Region: Where Do We Stand Today?

This review showed legislative action to regulate food marketing to children is very limited in the EMR. In the absence of comprehensive marketing legislation approaches, a few countries started implementing legislative actions through a stepwise approach and a few others have started planning for the change.

This report highlighted how the few legal reforms implemented have been mainly limited to local television and radio outlets. This finding identifies several challenges. First, despite the rise in the adoption of digital media and the displacement of legacy media, mobile applications and online outlets and products’ packaging have been frequently omitted from legal reforms (46). Yet, online platforms have been found to incorporate food and beverage advertisements of non-core food more than other marketing outlets (47). Digital media can be even more dangerous than television and radio outlets as it employs personal data collected on children’s behavioral patterns, interest, geolocation, etc. (48). It can hence exploit children using identified vulnerabilities. The limited focus on online outlets devices, print and packaging in legal reforms is not limited to the EMR; it’s a common gap identified worldwide (49). Second, limiting legislative measures and monitoring to local or national media channels creates a loophole that the food industry can take advantage of. As many EMR countries speak the same languages, advertisements on regional channels can influence the behavior of children across states’ borders, a phenomenon defined as cross-border marketing. Moreover, in the EMR, regional televisions have greater funding and influence than most national channels (22). At a WHO regional virtual meeting on childhood obesity in the EMR, stakeholders identified digital marketing as a challenging medium to target and cross-country collaboration as an important component to drive progress in reaching the WHO goals on marketing of unhealthy foods and beverages to children (50). Participants saw that the difficulty of integrating legal reforms on digital media should not stop them from initiating legal reforms on traditional forms of marketing—such as television, radio and print advertising and marketing in or around schools. Country representatives identified mapping of digital marketing in the region and receiving training on monitoring methodology on food marketing as pre-requisites to reaching regional goals (50).

The WHO EMR office assessed the adoption of nutrient profiling in years 2013, 2015, and 2017 (27). This study revealed a marked improvement in the adoption of nutrient profiling with 23% of states implementing them as a pre-requisite for marketing regulations. Moreover, adoption of reforms is likely to be associated with the countries’ political stability, Gross Domestic Product, and the level of government commitment. Indeed, countries in political instability like Djibouti, Iraq, Lebanon, Libya, Somalia, Somalia, Syria, and Yemen have not adopted any kind of restrictions. Food authorities are likely addressing the urgent crises in their territories. Countries that have taken actions toward regulating marketing of non-nutritious products are mainly middle- or high-income countries. This reflects the abundance of resources that these countries can allocate to combat childhood obesity compared to low-income states.

We learn from other countries around the globe that reliance on the industry’s self-regulation has yielded limited success. Even though the food industry funded reports suggest an impressive level of compliance with voluntary pledges, there exists a great deal of discrepancy with the results of scholarly articles and evaluations (51). Companies take advantage of loopholes to attract children to their products, implement lenient restrictions and assess compliance “mercifully” (52). An evaluation of products marketed to children in Canada revealed that 73% of unhealthy products were for companies which have committed to pledges on responsible marketing (53). Even though reliance on the industry’s self-regulation is not recommended, experts agree on the importance of collaboration with the private sector for the success of any change. Involvement of the industry and governmental entities in the regulatory design has been promoted by the theory of Responsive Regulation and has proven successful in nutritional legal reforms (54–57). As the risk of conflict of interest from engagement with the food industry is elevated, the WHO recommends implementation of safeguards to prevent and manage conflicts of interest in the area of nutrition, and this is important to protect against any involvement that may undermine governments’ efforts to protect children from marketing (58).

### Recommendations

The lack of overall progress to date, and the finding that no country in the EMR has implemented a comprehensive approach, suggest that the recommendations of the WHO 2018 report on implementation of marketing restrictions in the region remain largely relevant (22). EMR countries are urged to develop as comprehensive approach as possible on unhealthy food marketing to children, tackling both exposure and power. Countries are called on to form multisectoral working groups to develop regulation and to build legal capacity so that those responsible for drawing up the draft legislation can withstand potential legal challenges. Countries should include monitoring and evaluation processes to regularly assess if the implemented reforms are efficient in reaching desired goals. The WHO has encouraged countries to adopt an evidence-based nutrient profiling system to identify items covered by the marketing ban. If a comprehensive approach is not feasible, countries were advised not to delay their intervention and to implement a stepwise policy, focusing on the most popular media where children are exposed to marketing, until a comprehensive approach becomes feasible. Finally, regional collaboration and cooperation are vital to safeguard youngsters from cross-border marketing (22).

### Limitations and Strengths

This study provides an overview from governmental and academic bodies on the status of EMR countries in reaching goals for marketing legal reforms. Its strength is in filling an important literature gap and in employing a solid methodology. A systematic search on major databases and governmental websites was applied to yield a comprehensive overview on the subject. For completeness and since such legal reforms may not be found in scientific databases, the search was coupled with a survey of country representatives. The study has several limitations as well. Even though we employed a systematic search strategy, articles were not screened in duplicates. Moreover, data was missing from some countries due to lack of policy digitization and the lack of monitoring studies in these nations. Lastly, many countries lacked data on the extent of implementation of the legal reforms.

## Conclusion

To our knowledge, this is the first scholarly article that analyzes the regulatory framework of food marketing restrictions toward children in the EMR. Reinforcing the results of the 2018 WHO report (21), this study revealed that the road toward achieving the WHO recommendations for marketing of unhealthy foods and non-alcoholic beverages to children has been rarely traveled in the EMR. The majority of countries only discussed the WHO Recommendations and have not taken any legal action. Countries adopting legal reforms are doing so in a stepwise approach. Implemented reforms have been limited to the traditional media, leaving out the more influential media outlets. An analysis of the marketing media children in EMR countries are exposed to showed that most commercials are for unhealthy products even in countries where legislation is present. EMR states should coordinate among stakeholders within their countries and across the region to legislate food marketing policies in view of the heavy burden of childhood obesity and the impact of marketing on children’s dietary patterns.

## Author Contributions

AA-J and JJ: conceptualization, methodology, and writing—review and editing. JJ: investigation and writing—original draft preparation. Both authors have read and agreed to the published version of the manuscript.

## Author Disclaimer

The authors alone are responsible for the views expressed in this article and they do not necessarily represent the views, decisions, or policies of the World Health Organization or the other institutions with which the authors are affiliated.

## Conflict of Interest

The authors declare that the research was conducted in the absence of any commercial or financial relationships that could be construed as a potential conflict of interest.

## Publisher’s Note

All claims expressed in this article are solely those of the authors and do not necessarily represent those of their affiliated organizations, or those of the publisher, the editors and the reviewers. Any product that may be evaluated in this article, or claim that may be made by its manufacturer, is not guaranteed or endorsed by the publisher.
