# Supplementary material for: Marketing of Food and Beverages to Children in the Eastern Mediterranean Region: A Situational Analysis of the Regulatory Framework
Source: Front Nutr. 2022 May 18;9:868937. doi: 10.3389/fnut.2022.868937 (PMC9158545; doi:10.3389/fnut.2022.868937)
Supplement: Supplementary file 1 [file Data_Sheet_1.docx]

**Food Marketing to children and adolescents in the Eastern Mediterranean Region: A situational analysis of the regulatory framework**

Supplementary material: Questionnaire shared with focal points

1. Did your country discuss WHO recommendations for marketing of unhealthy food and nonalcoholic beverages to children? Highlight or leave the correct answer
2. Yes b. No c. I don’t know

If you answered yes:

1. In which year were the recommendations discussed?
2. Which ministries/entities discussed the recommendations?
3. Did the discussions involve the private sector? If yes, please describe how.
4. To the best of your knowledge, in your country, is it allowed to advertise for unhealthy food and beverage products? Please highlight your answer.

a) On children’s television channels: Yes No I don’t know

b) On children’s television programs: Yes No I don’t know

c) On radio stations: Yes No I don’t know

d) On the social media networks/digital media: Yes No I don’t know

1. Did your country adopt legislation that contributes to the implementation of the WHO recommendations on marketing of unhealthy food and beverages to children and/or adolescents?

Please describe date, name and description of the legislation.

Please describe which ministries/entities lead the implementation.

If you have a policy draft, feel free to indicate its name and describe it.

1. To the best of your knowledge, are there restrictions in place on the marketing of breast milk substitutes and infant formulae? If yes, please describe date, name and description of the legislation and which ministries/entities lead the implementation.
2. Did the private sector in your country perform any voluntary pledge (or self-regulatory mechanism) to regulate the marketing of unhealthy food products to children and/or adolescents? If yes, please describe the pledge (s) and as well as the entities involved.
3. To the best of your knowledge, are there any restrictions on the marketing of food in schools or other settings where children gather such as nurseries, sports clubs, after school clubs, etc.?
4. To the best of your knowledge, is there a national system in place allowing for the classification of food into healthy and less healthy categories (in reference to nutrition profiling)? If yes, please describe it.
5. Have there been any monitoring initiatives to assess the marketing of unhealthy products targeting children and adolescents? Example: a study assessing the percentage of advertisements of unhealthy products targeting children. These initiatives can be led by the government, private sector and/or academicians.
